# Maturation of Social-Vocal Communication in Prairie Vole (*Microtus ochrogaster*) Pups

**DOI:** 10.3389/fnbeh.2021.814200

**Published:** 2022-01-11

**Authors:** Megan R. Warren, Drayson Campbell, Amélie M. Borie, Charles L. Ford, Ammar M. Dharani, Larry J. Young, Robert C. Liu

**Affiliations:** ^1^Department of Biology, Emory University, Atlanta, GA, United States; ^2^Center for Translational Social Neuroscience, Yerkes National Primate Research Center, Atlanta, GA, United States; ^3^Department of Psychiatry and Behavioral Science, Emory University School of Medicine, Atlanta, GA, United States; ^4^Summer Opportunities of Academic Research Program, James T. Laney School of Graduate Studies, Emory University, Atlanta, GA, United States

**Keywords:** ultrasonic vocalization, maternal potentiation, sensory development, volitional, bioacoustics, social isolation, rodent

## Abstract

Impairments in social communication are common among neurodevelopmental disorders. While traditional animal models have advanced our understanding of the physiological and pathological development of social behavior, they do not recapitulate some aspects where social communication is essential, such as biparental care and the ability to form long-lasting social bonds. Prairie voles (*Microtus ochrogaster*) have emerged as a valuable rodent model in social neuroscience because they naturally display these behaviors. Nonetheless, the role of vocalizations in prairie vole social communication remains unclear. Here, we studied the ontogeny [from postnatal days (P) 8–16] of prairie vole pup ultrasonic vocalizations (USVs), both when isolated and when the mother was present but physically unattainable. In contrast to other similarly sized rodents such as mice, prairie vole pups of all ages produced isolation USVs with a relatively low fundamental frequency between 22 and 50 kHz, often with strong harmonic structure. Males consistently emitted vocalizations with a lower frequency than females. With age, pups vocalized less, and the acoustic features of vocalizations (e.g., duration and bandwidth) became more stereotyped. Manipulating an isolated pup's social environment by introducing its mother significantly increased vocal production at older (P12–16) but not younger ages, when pups were likely unable to hear or see her. Our data provide the first indication of a maturation in social context-dependent vocal emission, which may facilitate more active acoustic communication. These results help lay a foundation for the use of prairie voles as a model organism to probe the role of early life experience in the development of social-vocal communication.

## Introduction

Our understanding of the pathological and physiological development of social-vocal communication has improved through the use of traditional animal models such as rats and mice. For instance, variations in the acoustic features of mouse pup vocalizations offer insight into the emotional states of pups, while mouse models of neurodevelopmental disorders and early life stress display long-term changes in vocal behavior (Branchi et al., 1998; Liu et al., 2003; D'Amato et al., 2005; Kessler et al., 2011; Hernandez-Miranda et al., 2017). However, the translational utility of many rodent models is limited by their expressed social traits. Traditional rodent models fail to capture the breadth and diversity of human behavior wherein social communication is essential, such as biparental care of offspring and the ability to form long-lasting, selective social bonds (Bales et al., 2021).

Prairie voles (*Microtus ochrogaster*) have emerged as a valuable rodent model in social neuroscience due to their natural repertoire of social behaviors. Prairie voles typically form lifelong, socially monogamous relationships with a single mate, exhibit biparental rearing of pups, and engage in alloparental care (McGraw and Young, 2010; Sadino and Donaldson, 2018; Walum and Young, 2018). The neural mechanisms responsible for these behaviors are modulated by neuropeptides, including oxytocin, vasopressin, and dopamine (Nair and Young, 2006; Young et al., 2008; Bosch and Young, 2017; Walum and Young, 2018), which are important for social bonding and communication in many species (Albers, 2012; Oettl et al., 2016; Marlin and Froemke, 2017; Froemke and Young, 2021; Nagasawa and Kikusui, 2021), including humans. In fact, studies using prairie voles and other vole species have been vital in furthering our understanding of the neural basis of affiliative behaviors. However, despite the volume of neurobiological and ethological studies on prairie voles, the role of specific sensory modalities in social communication during affiliative behaviors is not well-understood. It is notable that audition occupies a disproportionally large portion of the prairie vole sensory cortex as compared to other rodents, occupying about twice as much space as the mouse auditory cortex (Campi et al., 2007; Krubitzer et al., 2011). As such, vocalizations may play a larger role in prairie vole social communication as compared to other rodents.

Prairie voles, like other rodents, emit ultrasonic vocalizations (USVs) above the range of human hearing during social interactions (Holy and Guo, 2005; Arriaga and Jarvis, 2013; Rieger and Marler, 2018). Adult males, similar to other rodent models (Chabout et al., 2012, 2015; Warren et al., 2021), modify their vocal activity based upon their social context (Lepri et al., 1988; Ma et al., 2014), increasing both the number and complexity of USVs in the presence of an unfamiliar female. Males do not, however, change their vocal behavior in the presence of a same-sex sibling (Ma et al., 2014). Females, in contrast, modify vocal emission when exposed to either a same- or opposite-sex social partner. However, both male and female voles increase their vocal output when injected with amphetamine, indicating that vocal emission by adult voles may be generally linked to appetitive conditions (Ma et al., 2014).

In general, young rodent pups are highly vocal and reliably emit isolation-induced vocalizations when outside the nest. Isolation USVs function to elicit maternal retrieval (Sewell, 1970; Shapiro and Insel, 1990; Brunelli et al., 1994; Ehret, 2005; Bowers et al., 2013; Schiavo et al., 2020). Mouse and rat pups that vocalize at high rates are retrieved more rapidly than less vocal pups (Bowers et al., 2013), while silent pups are not reliably retrieved (Zippelius and Schleidt, 1956; Brooks and Banks, 1973). Thus, these sounds provide a vital means of communication between pups and mothers. Interestingly, prairie vole pups emit high numbers of USVs when separated from their mother and this increase in USV emission is highly correlated with increased levels of stress hormones (Shapiro and Insel, 1990). Non-monogamous montane voles (*Microtus montanus*), in contrast, show slightly increased corticosterone levels when separated from their mothers but not a concomitant increase in vocal emission (Shapiro and Insel, 1990). Additionally, the features of vocalizations can be a readout of early-life experience. For instance, early-life maternal separation (P2–P10) in rat pups leads to modified vocal features (Kaidbey et al., 2019). Thus, USVs may provide a useful indicator of early-life social bonds, though the precise conditions that elicit their emission and the development of context-dependent emission are not well-understood.

In this study, we characterized the ontogeny of USVs emitted by prairie vole pups and assessed how they employ them as a means of communicating with their mother. Using a novel behavioral paradigm, we measured the vocal activity of pups from P6–P16, either in isolation or when their mother was present but physically separated. We characterized how vocalizations changed over the course of development as a function of sex, as well as the role of social context in modifying vocal emissions, and mapped these changes onto developmental milestones.

## Materials and Methods

### Subjects

Prairie vole pups (6–16 days after birth) were used to examine the ontogeny of social-vocal communication. 111 pups originating from 59 litters across 15 breeding pairs were used for behavioral experiments. A maximum of two pups per litter were recorded at the same age, and no pup was recorded more than once. Pups were returned to the parents after recordings to be used in other studies. All animals originated from a laboratory breeding colony that was derived from field-captured voles in Champaign, Illinois.

Animals were kept on a 14/10 h light/dark cycle at 68–72°F and 40–60% humidity with *ad libitum* access to food (Laboratory Rabbit Diet HF #5326, LabDiet, St. Louis, MO, USA) and water. Cages were filled with Bedo'cobbs Laboratory Animal Bedding (The Andersons; Maumee, Ohio) and contained environmental enrichment, including cotton pieces to allow nest building. Pups were kept in breeding cages with their parents until weaning at 20–23 days of age, then group-housed (2–3 per cage) with age-matched pups of the same sex.

All experiments were performed during the light cycle between 9 a.m. and 5 p.m. Experiments were conducted in strict accordance with the guidelines established by the National Institutes of Health and then approved by Emory University's Institutional Animal Care and Use Committee.

### Data Collection

All data collection occurred in a designated behavioral-recording room separate from the animal colony, with the home cage placed at the opposite side of the room where it did not generate sounds that could contaminate our recordings. To record isolation-induced ultrasonic vocalizations (USVs), individual pups (P6: *n* = 19; P8: *n* = 19; P10: *n* = 14; P12: *n* = 16; P14: *n* = 19; P16: *n* = 14. P = postnatal day, or days after birth, with P0 being the day of birth) were removed from their home cage and placed in a plexiglass recording chamber (14 × 16.5 × 14 cm) lined with clean Alpha-DRI bedding for 10 min while audio and video data were recorded. Approximately equal numbers of males and females were recorded at each age. Recordings began at P6 to minimize nest disturbances at very young ages. A subset of the pups, aged P8–P16 (*n* = 11, 11, 13, 12, and 12 for P8, P10, P12, P14, and P16, respectively) were also used to assess the effect of social context on pup-emitted vocalizations. Therefore, after the 10 min of isolation, pups' mothers were introduced into an adjacent, identical chamber for another 10-min recording. The two chambers were physically separated by a transparent plexiglass wall with a single hole in its center, 1 cm in diameter and 3 cm above the floor. During all recordings, animals had access to food (Laboratory Rabbit Diet HF #5326) and water gel (Clear H2O Scarborough; Scarborough, ME).

A microphone (Avisoft CM16/CMPA microphone) was placed into the pup's chamber to record audio data. Audio was sampled at 300 kHz (Avisoft-Bioacoustics; Glienicke, Germany; CM15/CMPA40-5V), and an UltraSoundGate (116H or 416H used for all recordings, Avisoft-Bioacoustics; Glienicke, Germany) data acquisition system was used and integrated with Avisoft-RECORDER software to store the data. A video camera (Canon Vixia HF R800) recorded a side-view video of the pup chamber at 30 fps.

In a set of control recordings, the same paradigm was used, but a second microphone was placed into the mother's chamber. The UltraSoundGate 416H was used to simultaneously record audio data from both microphones.

The datasets presented in this study can be found in online repositories. The name of the repository can be found in the data availability statement.

### Vocal Extraction

To extract vocal segments (continuous units of sound), audio files were processed with USVSEG, an open-source MATLAB-based USV detection and analysis program (Tachibana et al., 2020). Files were band-pass filtered between 15 and 125 kHz. Audio was characterized at a time step of 0.5 ms and sounds with fewer than 6 samples (corresponding to sounds shorter than 3 ms) were excluded. USVSEG was modified to produce a structure for each vocal segment, containing the time value of each sample, the frequency of sound at each sample, and the corresponding sound amplitude. This structure is referred to as a frequency contour. Due to the high number of harmonics (sounds at integer multiples of the lowest frequency tone) present in prairie vole pup vocalizations, USVSEG was also modified to allow up to seven unique sound frequencies to be extracted at each time point. The resultant structure was organized based on sound frequency at each sample, such that each contour contained a fundamental frequency contour tracing of the lowest frequency sound at each point, and separate traces for simultaneous sounds at higher frequencies.

Frequency contours were further refined using custom-written MATLAB scripts. Manual inspection of the data allowed us to determine optimal thresholds to filter out extracted noise such that >97% of extracted sounds were true vocalizations while only excluding 3% of vocalizations (data not shown; data verified by two trained viewers). Sounds with a median fundamental (lowest) frequency below 22 kHz were also excluded, as these most often corresponded to noise. Thus, all extracted USVs fell between 22 and 125 kHz. To further refine the extraction of vocal segments, successive sounds with instantaneous jumps in frequency (changes in sound frequency between two successive sound samples) exceeding 8 kHz or periods of silence exceeding 4 ms were separated into distinct segments. All analyses were conducted based upon the fundamental contour of each segment.

For experiments where two microphones were used, USVs were separately extracted from each microphone channel. Then, if a sound was picked up simultaneously on both microphones, the sound was attributed to the chamber on which the sound amplitude was greatest. This method was verified using sound playback from speakers, and side attribution accuracy was found to be >95%. The number of USVs emitted at each age did not significantly differ based on whether mother-emitted sounds were included (paired *t*-tests; −2.7 < *t* < −1.6, all *p* > 0.05; data not shown), so all USVs were attributed to the pup in the one-microphone recordings.

### Developmental Milestones

A separate cohort of pups (*n* = 10, 5 males and 5 females across 4 litters) was used to characterize the developmental trajectory of prairie vole pups. For these experiments, individual pups were recorded every 2 days from P6 to P16 to track their developmental trajectory (thus, each pup was recorded 6 times). For each recording, individual pups were removed from their home cage, their temperatures were measured via a PhysioSuite (Kent Scientific) temperature sensor, the pups were weighed, then placed into the plexiglass recording chamber. The chamber was lined with Alpha-DRI bedding and contained a food pellet and piece of water gel. Pups remained in the chamber for 10 min, after which their temperature was taken again, with temperature change used as a proxy for thermoregulation.

Starting at P6, a speaker was placed next to the open side of the recording chamber to assess functional hearing *via* an acoustic startle paradigm. Instead of using the plexiglass divider to seal the chamber, the open side of the chamber was enclosed using two stacked metal mesh blocks, as this would allow sound to efficiently propagate into the chamber. After a pup's second temperature reading, the pup was returned to the chamber and playback began on the speaker. The playback file consisted of 2 min of silence, followed by 50 ms of white noise. During playback, audio (Avisoft microphone, see above) and video (captured by Basler acA 1920-150uc camera) data were recorded to provide video confirmation of a startle response. Pups were assessed for a visible response to the noise, which consistently presented as the pup jumping. Playback occurred on every recording day from P6 until all pups within a litter (2–3 pups) startled.

Pups were also visually assessed at each age for ear and eye opening. Ear opening was defined as having a visible ear canal, and eye opening was defined as having the eyes fully open.

### Analyses

#### Converting Vocal Segments Into Vocalizations

**Figure 1:**
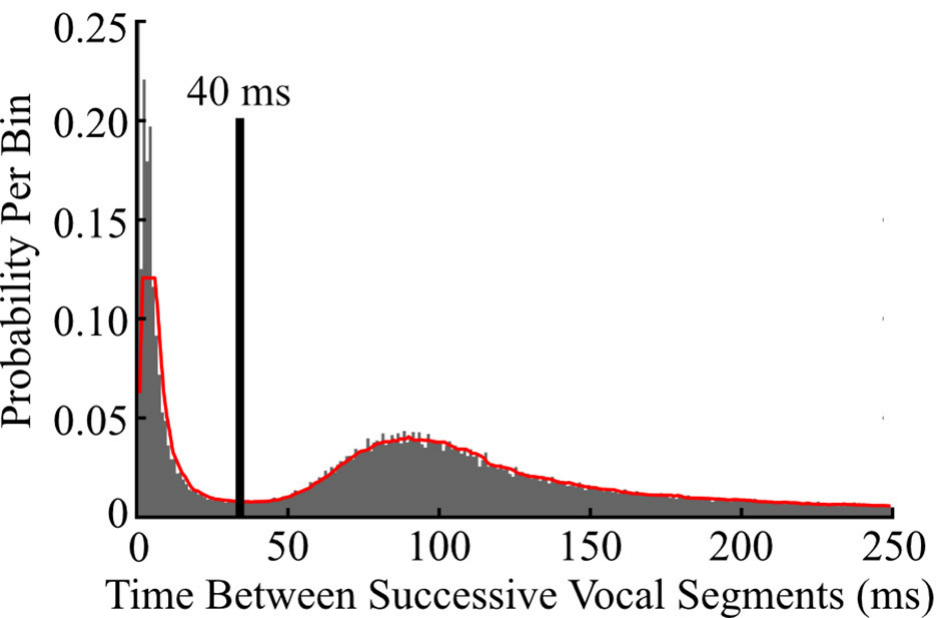
Determining a temporal threshold to consolidate successive vocal segments into vocalizations. To determine the optimal interval for collapsing successive vocal segments into vocalizations, a histogram of the time between all pairs of successive vocal segments (gray) was generated, smoothed (red trace), and the first local minimum (black line) was used as the consolidation threshold. Consecutive segments separated by 40 ms or less were subsequently consolidated into a single vocalization.

To determine thresholds for consolidating segments into vocalizations, a histogram of the silent intervals between successive segments was created and smoothed with a 5th-order one-dimensional median filter. The first local minimum was used as the threshold for consolidating two adjacent segments into a single vocalization (Figure 1). This consolidation threshold was then applied to the entire library of segments, yielding a library of vocalizations. The frequency contours of consolidated segments were unaltered during consolidation. Therefore, each vocalization in the library was described by the fundamental frequency contours of its component segments.

#### Quantifying Acoustic Features of Vocalizations

**Figure 2:**
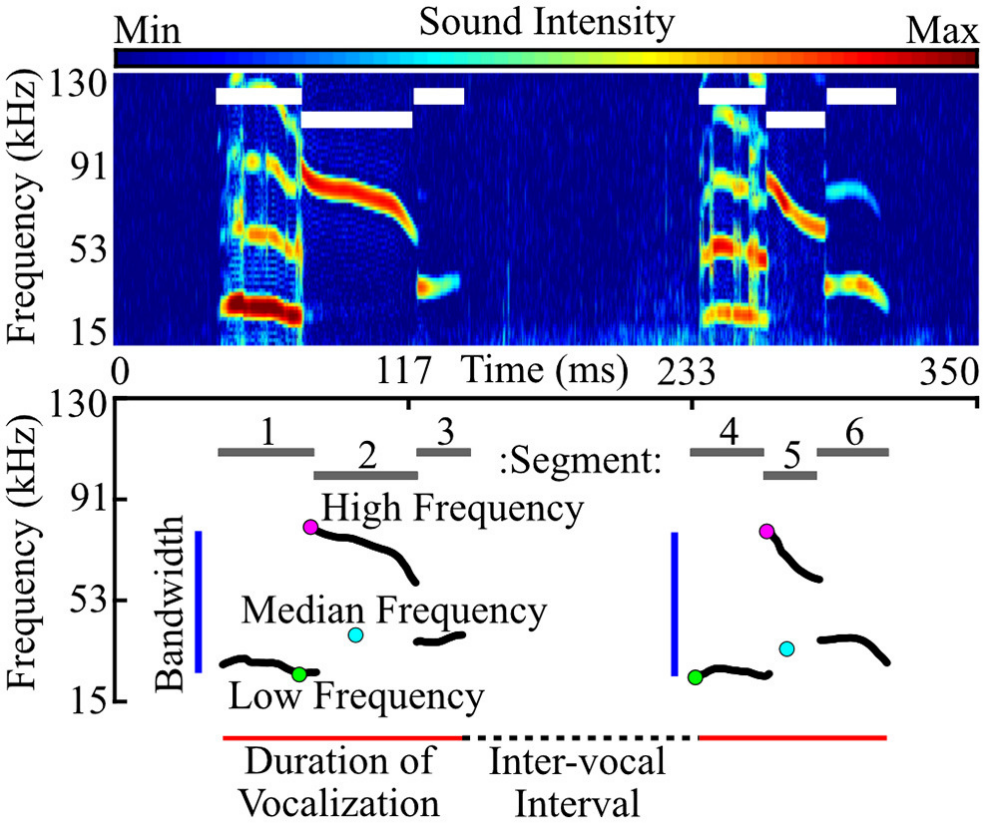
Vocal features were characterized by generating a fundamental frequency contour of each vocalization. Using recorded audio data (represented as a spectrogram; top), we extracted vocal segments, or continuous units of sound (white lines in top image show segment duration, corresponding to gray lines in bottom). Each segment was represented by its fundamental frequency contour (bottom; black lines), tracing the fundamental (lowest) frequency at each time point. Note that harmonics (frequencies at integer multiples of the fundamental) are not represented in the fundamental trace. Segments separated by 40 ms or less were then consolidated into vocalizations (bottom; red lines show duration), and the corresponding segment contours were collapsed to generate a single contour for each vocalization. Contours were used to characterize the acoustic features of each vocalization, as represented above. Inter-vocal interval is the time between two successive vocalizations. High, median, and low frequency are the highest, median, and lowest frequency within each fundamental contour, respectively. Bandwidth is the frequency range, or high frequency minus low frequency.

**Figure 3:**
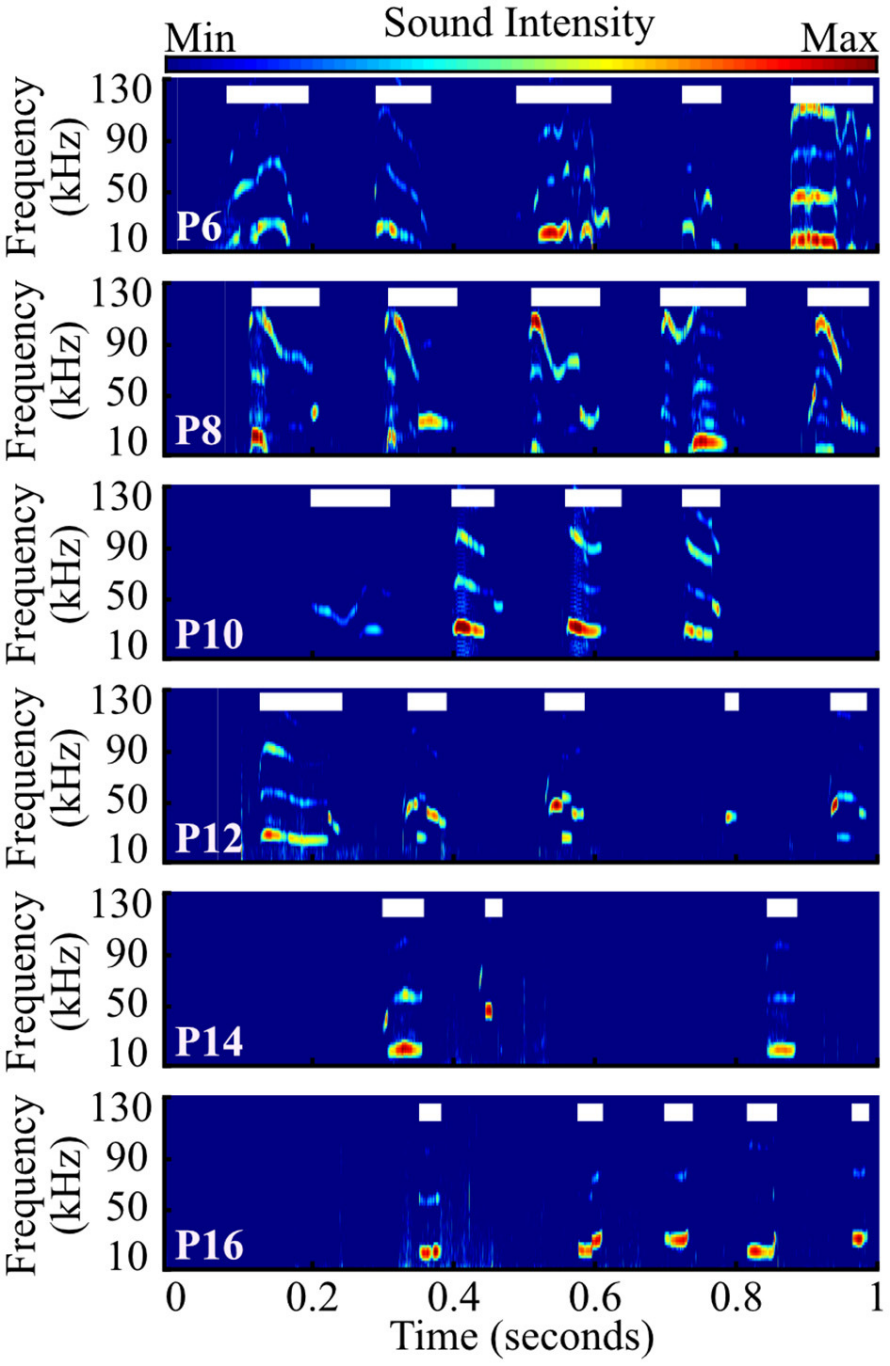
Representative examples of pup vocal activity across development. Example spectrograms contain one second of audio data recorded from prairie vole pups aged P6–P16. Each thick white line shows time when a single vocalization was extracted.

Fundamental contours were used to quantify the acoustic features of each vocalization (Figure 2). These features included duration (the end time minus the start time), median frequency, high frequency (the highest frequency across the fundamental contour), low frequency (the lowest frequency across the fundamental contour), bandwidth (the high frequency minus the low frequency), and slope (the average partial derivative across the contour). We also assessed the number of harmonics, sounds with frequencies falling approximately at integer multiples of the fundamental frequency (e.g., 1.8–2.2x, or 2.8–3.2x, etc.). Harmonics can be seen in Figures 2, 3. Harmonic sounds lasting fewer than 3 ms were excluded. The mode number of harmonics, or the most common number of harmonics across the entirety of the vocalization, was used to characterize harmonicity.

To control for the possibility of inflated significance with increased sample sizes (Bakan, 1966), as well as differences in the number of vocalizations emitted by individual pups, we compared vocal features at the level of individual animals. Thus, for each acoustic feature we found the average value across all vocalizations emitted by a single pup. This process was repeated for each pup and each feature, allowing individual animals to be represented by their own average value. Group differences in vocal features were then assessed as a function of both the age and sex of each pup. If a main effect of sex was not found, male and female data were collapsed. Assessment of vocal features across social context was conducted using the same method, except data were extracted separately for the 10 min of isolation vs. the 10 min when the mother was present.

#### Quantifying Vocal Emission Levels

Vocal emission levels were characterized multiple ways. First, to assess the impact of age on vocal rates, we counted the total number of vocalizations emitted by pups over the 10-min isolation period. To characterize changes in vocal emission as a function of social context, the number of vocalizations emitted while the mother was present was also counted. Lastly, to assess vocal emission on a finer timescale, we also determined the number of vocalizations emitted by pups in each minute of the audio recording.

#### Quantifying Variability in Vocal Features

To quantify the level of variability in individual acoustic features, we first extracted the feature of interest (e.g., bandwidth) from all vocalizations emitted by a single pup. We then calculated the variance (sum of squares divided by the number of vocalizations minus 1) of that feature. This analysis was repeated for each pup and each feature of interest (duration, bandwidth, and number of vocal segments per vocalization).

### Statistics

Data are shown as mean ± standard deviation unless otherwise indicated. Comparisons between ages and sexes were done with two-way ANOVAs. If a main effect of sex was not found, data were collapsed across sex and then analyzed with a one-way ANOVA. All one-way ANOVAs were combined with a *post-hoc* Tukey's HSD to correct for multiple comparisons. Comparisons of changes in temperature over time used one-tailed *t*-tests to determine whether group distributions fell significantly below zero (indicating a lack of thermoregulation). The vocal emission of individual pups recorded across contexts was compared with a mixed factorial ANOVA, then pairwise comparisons were conducted via paired two-tailed *t*-tests with a Benjamini-Hochberg *post-hoc* correction. The number of vocalizations emitted per time bin was averaged across context, and then the values were compared with a paired *t*-test. Changes in acoustic features across contexts were assessed via z-tests, comparing the distributions of differences to zero, or no difference. A mixed factorial ANOVA was used to compare changes in vocal emission across social context phases for different ages, with a *post-hoc* Benjamini-Hochberg test conducted solely on paired *t*-tests comparing vocal emission across context within each age group. All alpha values were set to 0.05. All statistical analysis was performed with MATLAB (Mathworks).

## Results

### Older Pups Emit Fewer Isolation-Induced Vocalizations Than Younger Pups

**Figure 4:**
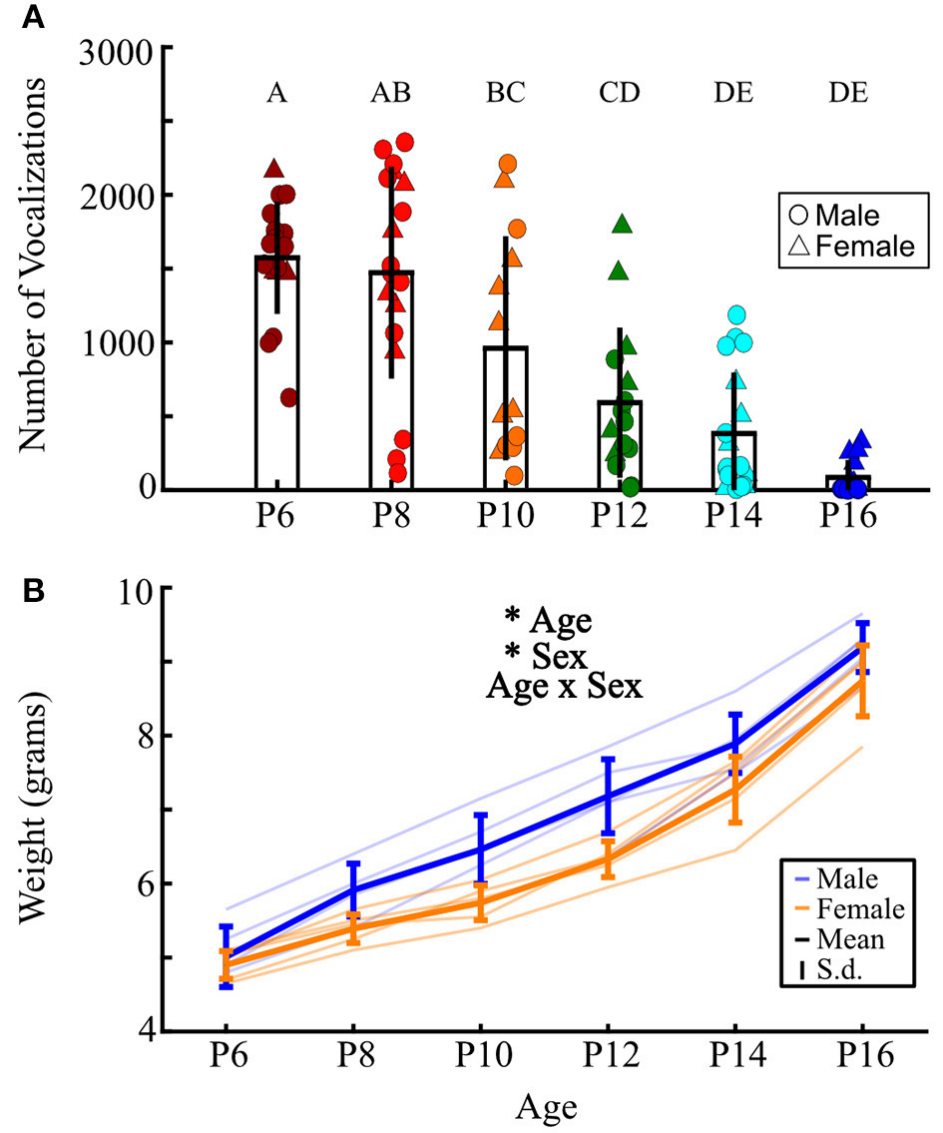
Older pups emit fewer isolation-induced vocalizations than young pups. **(A)** Each point represents the total number of vocalizations emitted by a single pup across 10 min of isolation; males are circles, females are triangles. Data presented as mean ± 1 standard deviation. *n* = 19, 19, 14, 16, 19, and 14 for P6, 8, 10, 12, 14, and 16, respectively. There was no main effect of sex (two-way ANOVA, age-by-sex), so male and female data were collapsed. Ages sharing a letter do not significantly differ from each other (*p* ≥ 0.05, Tukey's HSD *post-hoc* correction), while all other comparisons are significantly different (*p* < 0.05). **(B)** Weights of pups across development. Each thin line represents an individual pup recorded over time. Thick blue lines show mean ± 1 s.d. for males, orange show females. Two-way ANOVA found a main effect of age and sex (all *p* ≤ 10^−3^), with no significant interaction. *p < 0.05.

We recorded 84,294 isolation-induced vocalizations from 101 prairie vole pups, distributed over sex and ages P6–P16 (Figures 3, 4). Males and females emitted similar numbers of vocalizations [*F*_(1, 84)_ = 2.39, *p* = 0.13, two-way ANOVA], so count data were collapsed across sex. We found that older pups emitted significantly fewer vocalizations than younger pups [Figure 3; *F*_(5, 90)_ = 21.43, *p* <10^−10^; one-way ANOVA; Figure 4]. P6 pups emitted significantly more vocalizations than pups from P10 to P16 (all *p* ≤ 0.02; Tukey's HSD), as did P8 pups compared to those aged P12–P16 (all *p* ≤ 10^−5^), and P10 pups compared to P14 and P16 pups (all *p* ≤ 0.038). Thus, similar to other rodent species (Naito and Tonoue, 1987; Campbell et al., 2014; Johnson et al., 2017) and prior reports in prairie voles (Rabon Jr et al., 2001; Terleph, 2011), pup vocal emission decreases with age.

### Acoustic Features Differ by Sex and Across Development

Considering all vocalizations regardless of age, USVs had median frequencies of 33 (±8 kHz standard deviation) for males and 34 kHz (±9 kHz) for females. The average range of male frequencies fell between 28 and 42 kHz (±8 and 14 kHz), while female vocalizations spanned 27 and 45 kHz (±8 and 16 kHz, respectively). 82.2% of prairie vole USVs contained at least one harmonic component, and 24.9% contained at least 3 harmonics, in contrast to USVs of other rodents like mice, which typically have no harmonics. Hence, prairie vole pups emit vocalizations with different spectral characteristics than other traditionally used rodents.

**Figure 5:**
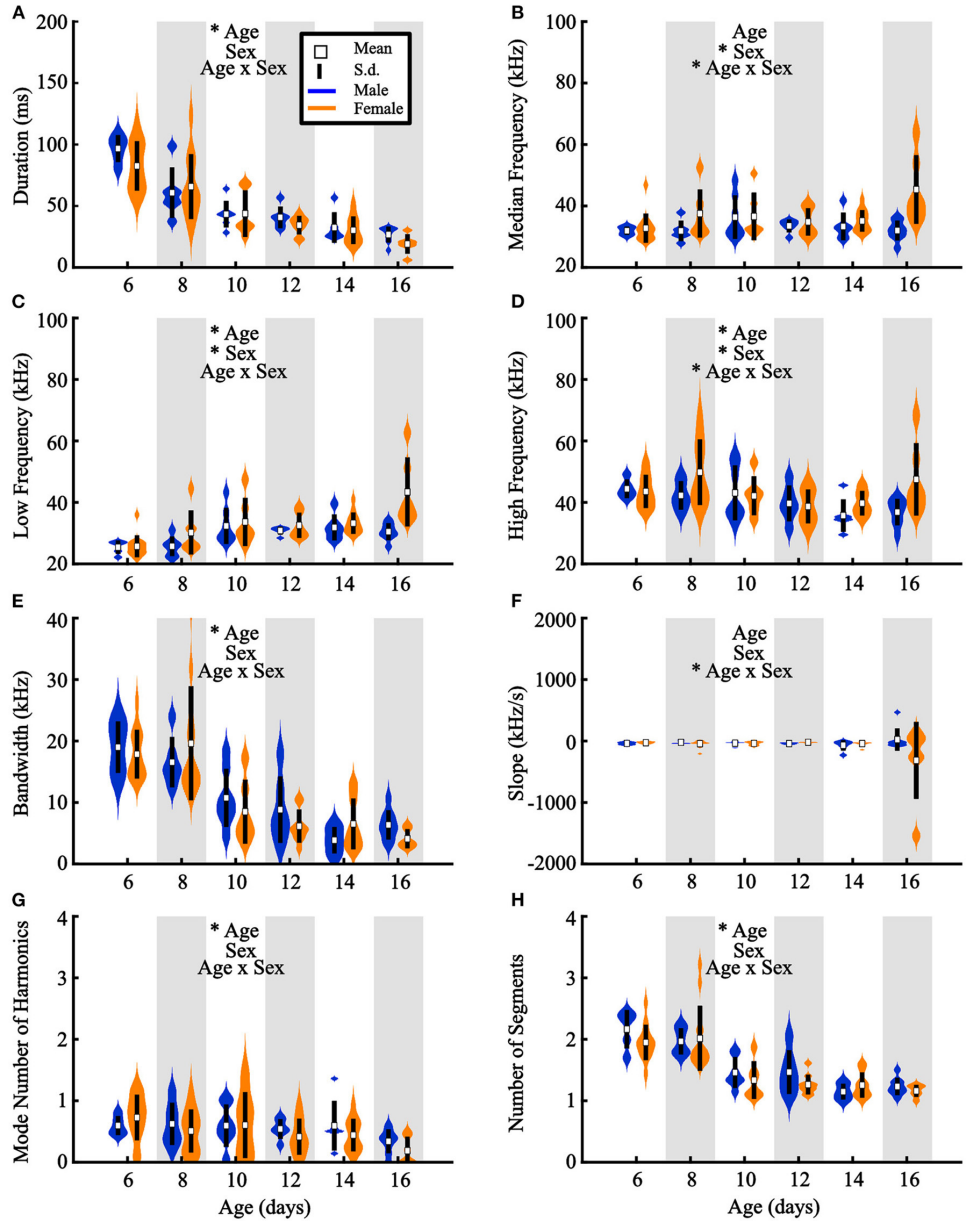
Acoustic features of pup vocalizations differ across development. Individual pups are represented by the average value across all vocalizations emitted by that pup. Violin plots represent feature distributions for **(A)** duration, **(B)** median frequency, **(C)** low frequency, **(D)** high frequency, **(E)** bandwidth, **(F)** slope, **(G)** number of harmonics, and **(H)** number of segments per vocalization, sorted by age and sex. Wider locations indicate a higher number of animals exhibiting that average value. *Age = main effect of age, *Sex = main effect of sex, *Age x Sex = significant interaction term (two-way ANOVA). White squares represent group means and vertical black lines represent ± 1 standard deviation. Males are presented in blue, females in orange. *p < 0.05.

Across development, pups across species typically exhibit acoustic changes in their vocalizations (Liu et al., 2003; Yurlova et al., 2020). For prairie vole vocalizations, we ran two-way ANOVAs assessing differences in acoustic features as a function of both sex and age (see section Methods, Figure 2). Females emitted vocalizations that were significantly higher in low [*F*_(1, 84)_ = 10.39, *p* = 0.002], median [*F*_(1, 84)_ = 9.94, *p* = 0.002], and high frequency [*F*_(1, 84)_ = 4.76, *p* = 0.032] than males (Figures 5B–D). Interestingly, we found in a separate cohort that males also weighed significantly more than females {Figure 4B; two-way ANOVA, main effect of age [*F*_(5, 47)_ = 119.7, *p* <10^−5^] and sex [*F*_(1, 47)_ = 25.43, *p* <10^−5^]}, suggesting a size difference might contribute to differences in vocal frequencies. Regardless, in agreement with our analysis-by-vocalization, our analysis-by-individual confirmed that the frequency distribution of male vocalizations is downshifted from that of their female littermates.

We next observed a significant main effect of age in six assessed vocal characteristics (Figure 5). Older pups emitted vocalizations with shorter durations [*F*_(5, 84)_ = 34.34, *p* <10^−10^], lower high frequencies [*F*_(5, 84)_ = 3.07, *p* = 0.01], higher low frequencies [*F*_(5, 84)_ = 8.17, *p* <10^−10^], lower bandwidths [*F*_(5, 84)_ = 23.09, *p* <10^−10^], fewer harmonics [*F*_(5, 84)_ = 2.53, *p* = 0.04], and fewer segments [*F*_(5, 84)_ = 9.43, *p* <10^−10^] than younger pups.

Additionally, three acoustic features exhibited a significant age-by-sex interaction: median frequency [*F*_(5, 84)_ = 2.69, *p* = 0.03], low frequency [*F*_(5, 84)_ = 2.78, *p* = 0.02], and slope [*F*_(5, 84)_ = 2.59, *p* = 0.03] (Figure 5). Thus, along with the decrease in vocal emission at older ages, our results indicate that isolation-induced USVs become shorter, simpler in structure, and less frequent as pups age, defining a developmental trajectory of prairie vole pup vocal emission from P6.

### Acoustic Features of Vocalizations Become More Stereotyped With Age

**Figure 6:**
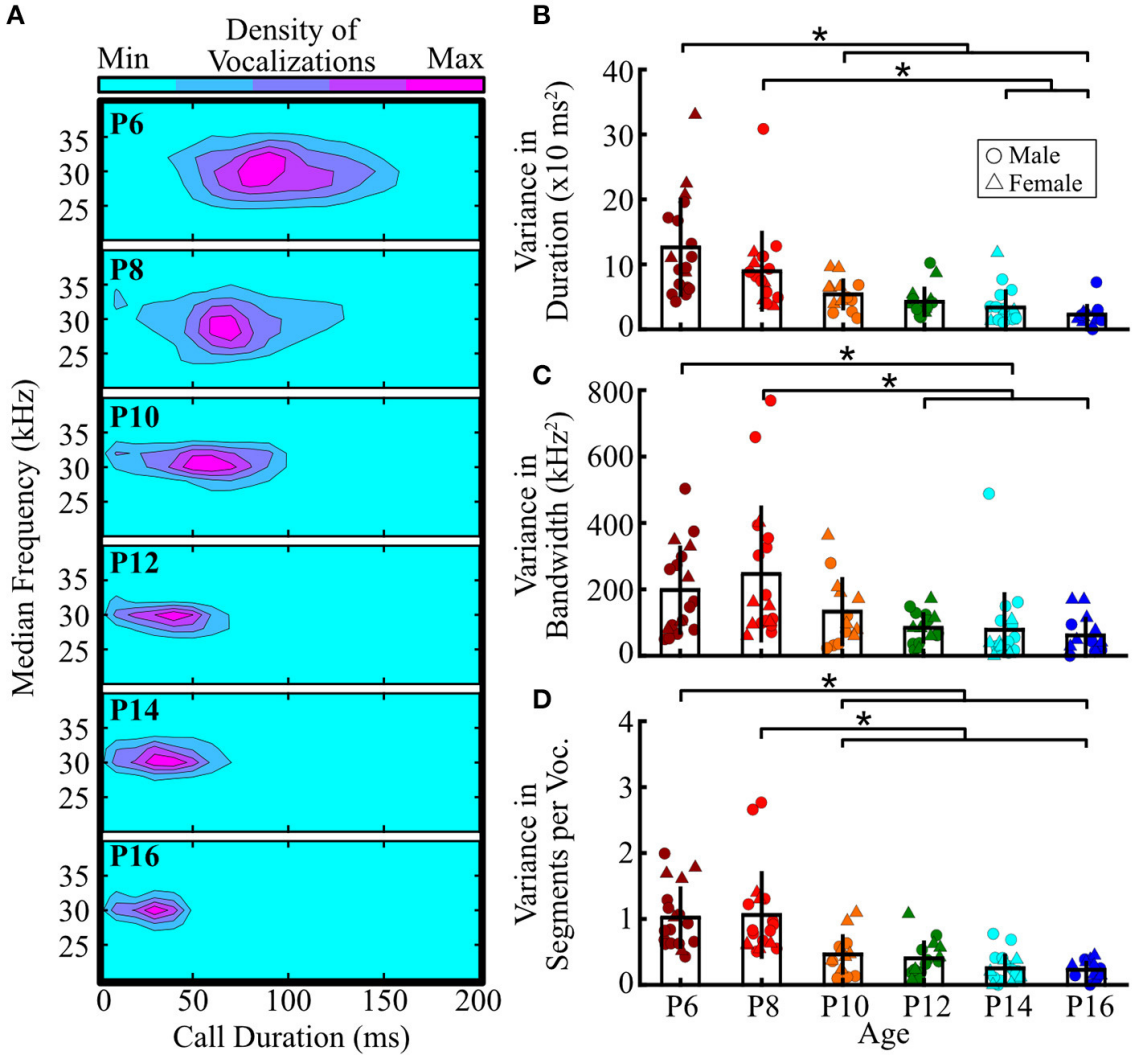
Pup isolation vocalizations become more stereotyped with age. **(A)** Distributions of median frequency and duration of pup vocalizations across ages. Warmer colors indicate greater numbers of vocalizations falling within the region. **(B–D)** Within-animal variability across ages for **(B)** vocal duration, **(C)** bandwidth (high minus low frequency of the fundamental contour), and **(D)** number of segments per vocalization. Each dot represents the variability for a single pup, measured by finding the variance of that feature (sum of squares/n-1) across all calls emitted by that pup, with higher values indicating more variability. There was no main effect of sex for any comparison, so male and female data were collapsed. Bars represent group means, and vertical lines represent standard deviations. Males are represented by circles, females by triangles. *p < 0.05 (ANOVA with Tukey's HSD *post-hoc* correction).

One intriguing feature of pup vocalizations is that for many acoustic features, the distributions seemed narrower for older pups, indicating a potential change in the stereotypy of vocal features with age (Figure 6A). Therefore, we next assessed age-related differences in the acoustic variability of duration, bandwidth, and the number of segments per vocalization – collapsing data across sex [no main sex effects, 0.4 ≤ *F*_(1, 84)_ ≤ 3.0, all *p* ≥ 0.09; two-way ANOVA]. We found that the range of vocal durations significantly decreased with age [F_(5, 84)_ = 9.1, *p* <10^−5^; one-way ANOVA; Figure 6B]. P6 pups emitted vocalizations with significantly greater variability in duration than pups aged P10 to P16 (all *p* ≤ 0.01; Tukey's HSD), as did P8 pups compared to P14 and P16 pups (all *p* = 0.01). Therefore, not only do vocalizations become shorter as pups age, but vocal duration also becomes more consistent.

Vocal bandwidths also became more stereotyped with age [*F*_(5, 84)_ = 4.67, *p* < 0.001; one-way ANOVA; Figure 6C]. P6 pups emitted vocalizations with significantly greater variability in bandwidth than P14 pups (*p* < 0.05, Tukey's HSD), as did P8 pups relative to those aged P12–P16 (all *p* < 0.02). P10 pups, however, had intermediate values that did not significantly differ from pups at any other age. Thus, pups emit vocalizations with the greatest variability in bandwidth at young ages, and the bandwidth gradually becomes more stereotyped with age.

Lastly, we assessed changes in variability in the number of segments per vocalization and found that younger pups showed the greatest variability in the number of segments within individual vocalizations [*F*_(5, 84)_ = 10.5, *p* <10^−7^; one-way ANOVA; Figure 6D]. Both P6 and P8 pups emitted vocalizations that were significantly more variable than P10–P16 pups (all *p* ≤ 0.02). Taken together, these results indicate that the acoustic features of prairie vole pup vocalizations, including vocal duration, bandwidth, and the number of unique sound units per vocalization, become more stereotyped at older ages. Thus, not only do the overall acoustic features of pup vocalizations change with age, but the feature distributions also narrow, indicating a potential reliance on more standardized vocalizations at older ages.

### Pups Modulate Vocalizations in an Age and Context-Dependent Fashion

**Figure 7:**
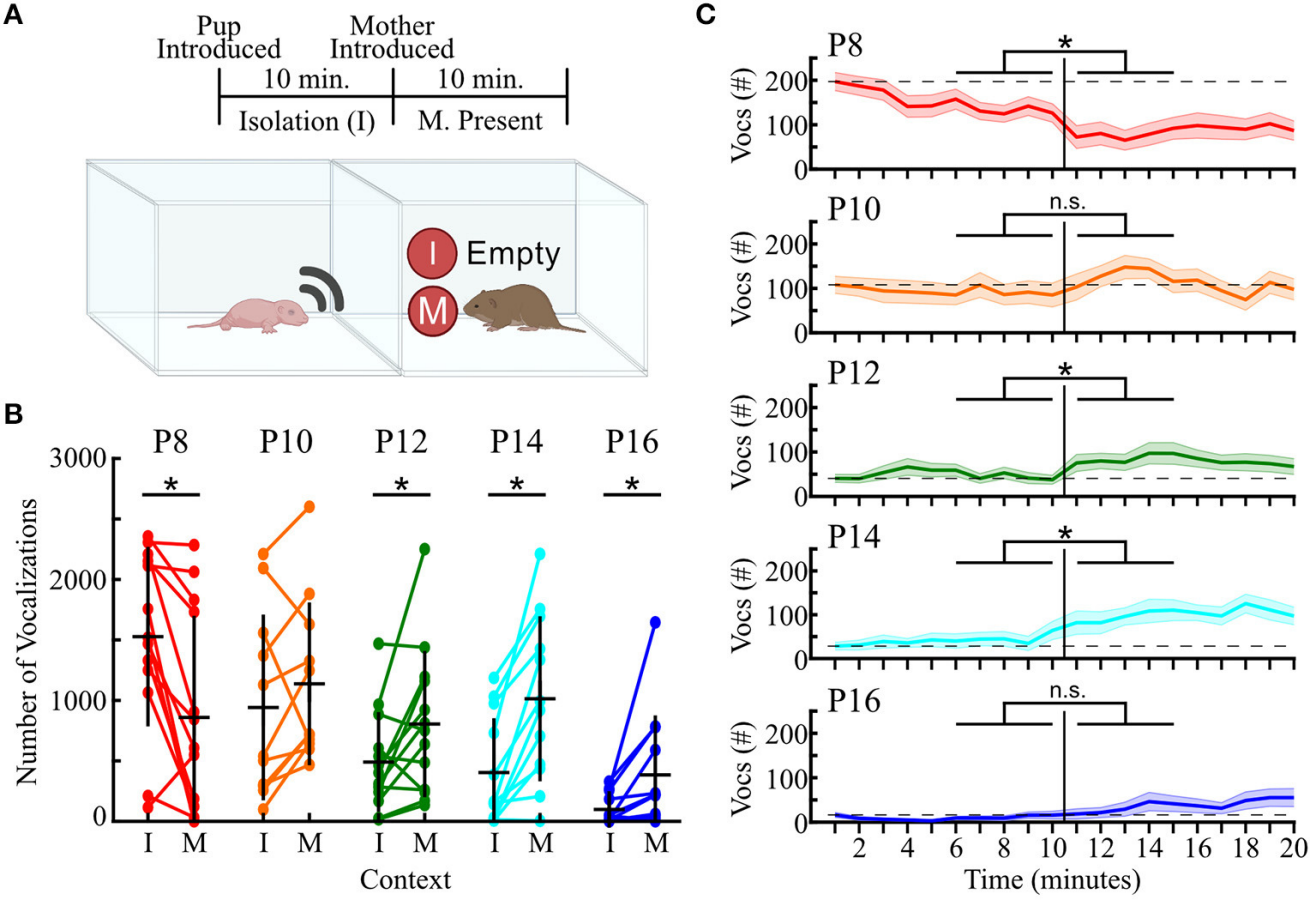
Vocal responses to social context change with pup age. **(A)** Timeline (top) and schematic (bottom) of the two recording contexts. I, isolation; M, mother present. Pup was audio- and video-recorded in each context. **(B)** Dots indicate the number of calls emitted while a pup was in isolation (left; I) or while their mother was in an adjacent but physically distinct chamber (right; M), with lines connecting the same pup across contexts. *n* = 11, 11, 13, 12, and 12 for P8, 10, 12, 14, and 16, respectively. Horizontal black lines represent group means, and vertical black lines represent standard deviations. Mixed factorial ANOVA indicated a main effect of age and social context, and a significant age-by-context interaction (all *p* ≤ 0.001). Then paired *t*-tests compared contexts within age. *Significant comparison after a Benjamini-Hochberg *post-hoc* correction. **(C)** Number of vocalizations emitted by pups in each 1-min bin over the 20-min recording. Colored horizontal lines indicate mean values across animals, with shaded areas representing ± 1 SEM. Vertical black line indicates time when the mother was introduced. Horizontal dashed line shows the average number of USVs emitted during the first minute of isolation. Colors as in **(B)**. *p < 0.05 when comparing the total number of vocalizations in the final 5 min of isolation (left) vs. the first 5 min after the mother was introduced (right) across all pups (paired *t*-test with Benjamini-Hochberg *post-hoc* correction for multiple comparisons).

In other rodent models, ultrasonic vocalizations are thought to act as a means of social communication, with adult animals modifying the rate of emission based upon social context (Whitney et al., 1974; Nyby et al., 1979; Whitney and Nyby, 1979), and isolated pups using USVs to elicit retrieval responses from adults (Zippelius and Schleidt, 1956; Sewell, 1970). Therefore, we next aimed to determine whether prairie vole pups modify their vocalizations as a function of their social context, and whether any social-context dependent changes differ as a function of age. To this end, after the isolation, a subset of pups aged P8–P16 (*n* = 59) had their mother introduced into a chamber that was attached to the pup's recording chamber (Figures 7A,B).

We found that introduction of the pup's mother led to different effects on vocal activity based on pup age. A mixed factorial ANOVA (age-by-context) uncovered a main effect of age [*F*_(4, 56)_ = 5.34, *p* <10^−4^] and social context [*F*_(1, 56)_ = 5.35, *p* = 0.02], as well as a significant age-by-context interaction [*F*_(4, 56)_ = 11.95; *p* <10^−6^]. At P8, introducing the mother significantly decreased pup vocal output, with pups dropping from an average of 1,527 ± 742 vocalizations in isolation to only 859 ± 841 vocalizations with the mother present [*t*_(13)_ = 3.72, *p* = 0.003, paired *t*-test]. In contrast, pups aged P12–P16 significantly increased their vocal emissions (all *p* ≤ 0.03). P10 was the only age group that did not show a significant change in vocal emission when transitioning from isolation to the mother-present condition [*t*_(10)_ = −1.37, *p* = 0.20]. Taken together, these results provide the first indication of a developmental maturation in the social context-dependent vocal emission of prairie voles.

To determine whether introducing the mother led to rapid changes in vocal activity, we next compared the total number of vocalizations emitted in the 5 min directly preceding the introduction of the mother to the 5 min directly following (Figure 7C). At P8, we found a significant reduction in the number of USVs between these periods (paired *t*-test, Benjamini-Hochberg *post-hoc* correction; *p* <0.002). In contrast, no significant difference existed for P10 animals. P12 and P14 pups, however, exhibited a significant increase in vocal emission upon introduction of the mother (*p* <0.02). P16 pups did not show a significant modification in vocal emission (*p* = 0.11), but the number of USVs emitted by P16 animals was lower than at any other age point. Therefore, this lack of significance may be due to low emission levels, as we do see a significant difference if we expand our temporal window to 10 min (Figure 7B). Overall, these data indicate that introduction of the mother leads to a rapid and lasting change in the vocal emission levels of prairie vole pups that had just endured a short-term social isolation.

### Acoustic Features of Vocalizations Do Not Differ Across Social Contexts

**Figure 8:**
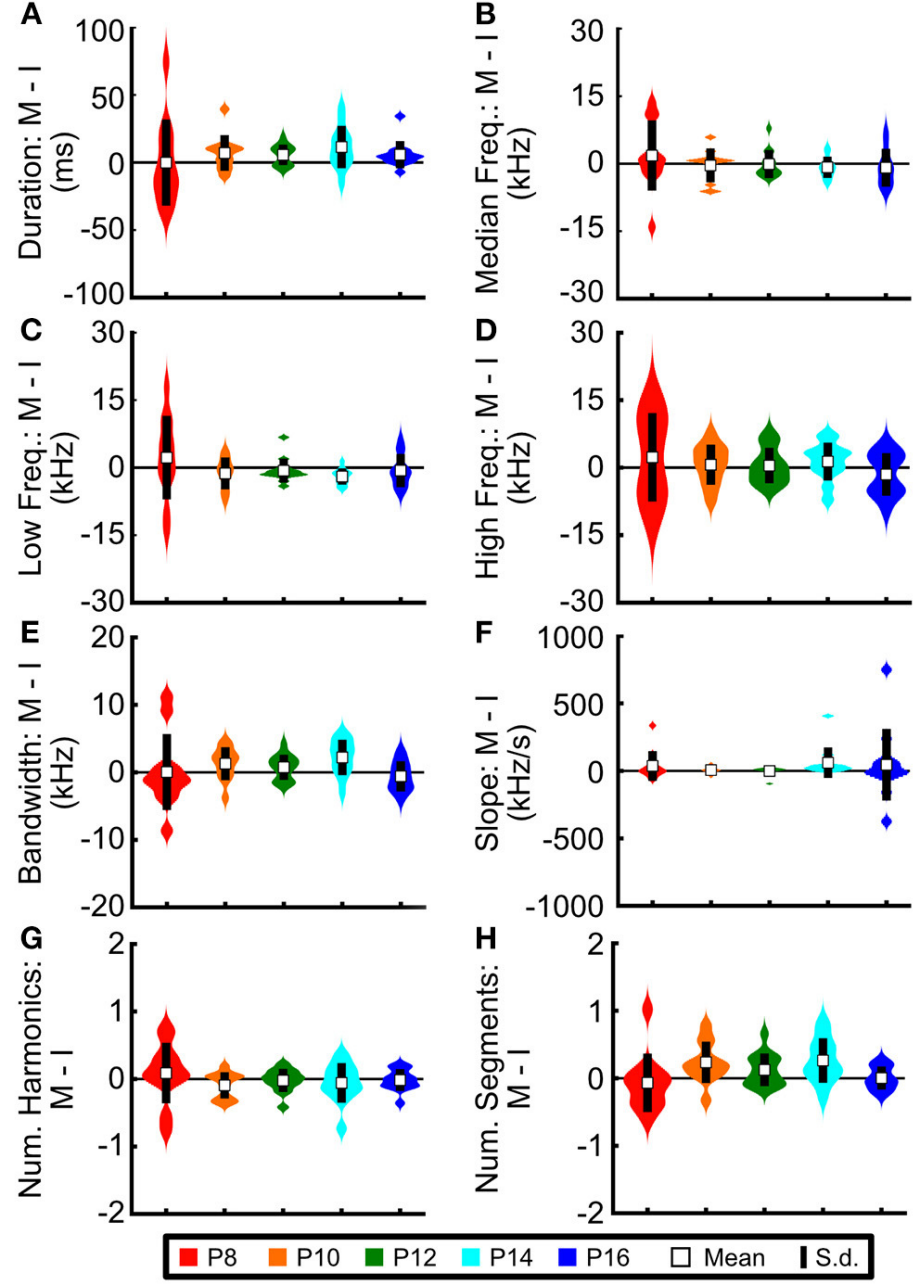
Prairie vole pups do not modify the acoustic features of USVs across social contexts. Difference in average acoustic features for individual pups between the isolated context and the mother-present context for **(A)** duration, **(B)** median frequency, **(C)** low frequency, **(D)** high frequency, **(E)** bandwidth, **(F)** slope, **(G)** number of harmonics, and **(H)** number of segments per vocalization, calculated by subtracting the average value of each individual pup in the isolated context (I) from the same pup's average value during the mother-present condition (M). No distributions differed significantly from zero (z-test, all z ≤ 1.09).

Other rodent species show changes in the acoustic features of their vocalizations based upon their social context (Chabout et al., 2012, 2015; Warren et al., 2021). Therefore, we next aimed to determine whether this also held for prairie vole pups. In fact, no feature showed significant differences across context (Figure 8; all z-scores ≤ 1.09). Thus, while pups modulate the number of vocalizations emitted based upon social context, context alone is not sufficient to modify those vocalizations' acoustic features.

### Context-Dependent Vocal Changes Are Preceded by Key Developmental Milestones

**Figure 9:**
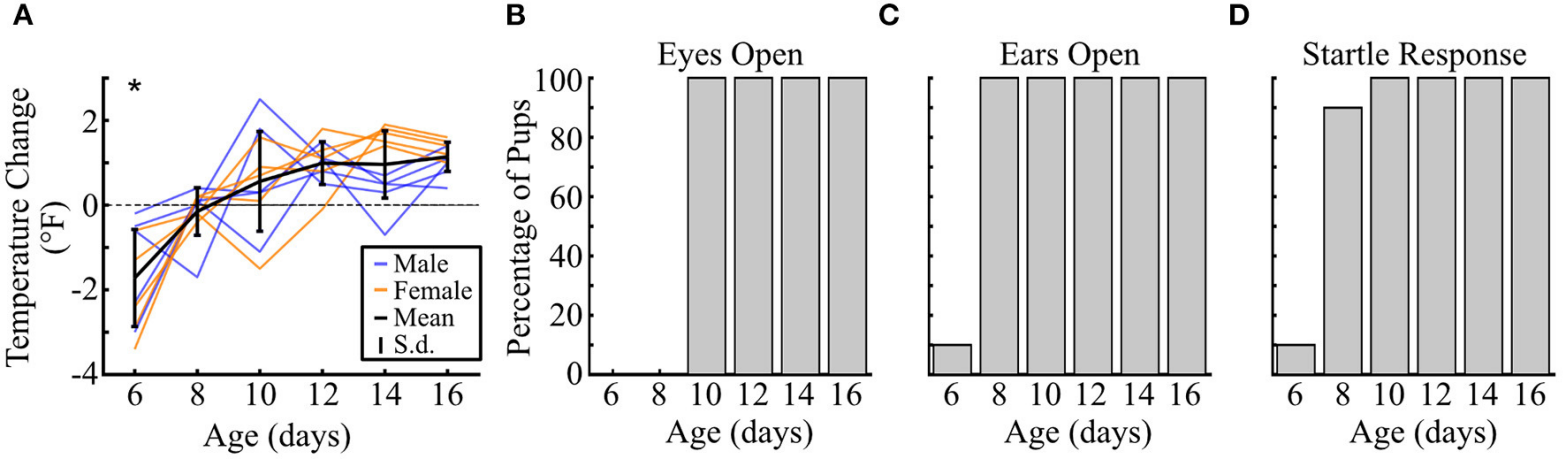
Prairie vole pup developmental timeline. **(A)** Change in temperature of individual pups after 10 min of isolation. Negative values indicate a decrease in temperature. Colored lines represent individual animals over time, with males in blue (*n* = 5) and females in orange (*n* = 5). Solid horizontal black line indicates mean value, with vertical lines showing standard deviation. Dashed line shows location of 0, or no change in temperature. *p < 0.05, distribution compared to zero with a one-tailed *t*-test. **(B)** Bar plots show the percentage of pups at each age **(B)** with open eyes, **(C)** with open ears, and **(D)** exhibiting a startle response to a loud sound.

One core need experienced by many rodent pups is body temperature maintenance, and temperature decreases have been associated with increased USV production in other rodents (Okon, 1970; Hofer and Shair, 1992). Vocal emission may be linked to the process of generating heat itself (Blumberg and Alberts, 1990; Hofer and Shair, 1993) or simply serve as a communication signal to mothers for retrieval back to the warmth of the nest (Ehret, 2005). The onset of thermoregulation is therefore a vital developmental milestone that could influence whether and how much vole pups vocalize when isolated. To assess this possibility, we used a separate cohort of pups (*n* = 10) to longitudinally track developmental markers every 2 days from P6 to P16 (Figure 9). We found that pups could not maintain their temperature across 10 min at P6 [*t*_(9)_ = −4.50, *p* <10^−3^, one-tailed *t*-test], but were able to do so from P8 onwards (all *p* ≥ 0.22; Figure 9A). Hence, over the age range where we observed changing context-dependent USV emission, the need to thermoregulate during isolation was likely not a major factor in driving this vocal modulation.

Another documented driver of pup USV emission in some rodent species is brief maternal reunion and contact, which potentiates USV calling if the mother is subsequently removed (Hofer et al., 1998; Shair, 2014). In our paradigm, unlike standard maternal potentiation paradigms, pups could not physically contact the mother. Instead, pups could likely use auditory or visual cues to recognize her presence. We therefore checked when these sensory modalities were developmentally functional. For audition, a single pup's ears were open at P6, while all other pups' ears opened between P6 and P8 (Figure 8B). Ear opening effectively corresponded with the presence of functional hearing, as confirmed through an acoustic startle paradigm. Hence, the ability to hear the mother on the other side of the barrier was likely possible before P10. Meanwhile, eye opening did not occur until between P8 and P10. Thus, by P12 when the addition of the mother significantly increased the number of vocalizations emitted, vole pups would have already passed key sensory developmental milestones that could enable better sampling of changes in their social environment.

## Discussion

Our study demonstrates a developmental maturation in both the features of prairie vole pup vocalizations and the role that social context plays in modifying vocal emission. Specifically, we found that older prairie vole pups emit fewer vocalizations that are simpler and more stereotyped than younger pups. Additionally, and more importantly, how pups vocally respond to changes in their social context switches around P10. Whereas, young pups decrease their vocal activity when their mother is present but physically unattainable and potentially unnoticed, older pups who can sense the mother's presence increase their vocal emissions. Interestingly, this change in vocal emission is not accompanied by changes in the vocalizations' acoustic features. Together, these results define a developmental trajectory for the use of stereotyped pup USVs as a means of social communication, opening the door for leveraging prairie vole pup USVs as a developmental readout of social motivation and affiliative behavior.

To our knowledge, this work provides the first report of a sex difference in the acoustic features of prairie vole pup USVs. Vocalizations as a whole centered around 32 kHz, in line with previous work (Colvin, 1973). However, the frequency content of male pups was downshifted from age-matched females. This finding is consistent with work in rat pups showing that females emit higher frequency vocalizations on average compared to males. Since females were consistently smaller than males, we considered whether size-frequency allometry—hypothesized (Morton, 1977) and found (Ey et al., 2007; Bowling et al., 2017) to explain differences in the fundamental frequency of harmonically structured vocalizations across primate and carnivore species—might explain our results. While pup weight significantly increased over time, our data showed no significant effect of age on the median frequency of vocalizations. In fact, low frequency significantly increased with age, opposite of what a size-frequency allometry might predict. This discrepancy likely reflects the fact that rodent USVs are probably generated by a different mechanism (Roberts, 1975; Mahrt et al., 2016; Riede et al., 2017) than the vocal fold oscillations of larger species. The presence of a size-independent sex difference should therefore be factored into models of rodent vocal emission.

Acoustic variability is reduced over vocal development in several species that learn to refine their vocalizations with experience [e.g., humans (Lee et al., 1999), birds (Ölveczky et al., 2011), and bats (Fernandez et al., 2021)]. We found that prairie voles, a presumed vocal non-learning species, exhibited a similar pattern. Across development, pup vocalizations became shorter, had narrower bandwidths, contained fewer harmonics, and had fewer segments [corresponding to previous findings (Terleph, 2011)]. Additionally, vocalizations became more stereotyped in duration, bandwidth, and the number of segments per vocalization with age. Another vocal non-learning species, the laboratory mouse [*Mus musculus* (Hammerschmidt et al., 2012; Arriaga and Jarvis, 2013; Mahrt et al., 2013)], also emits vocalizations that become more stereotyped with age – narrowing in frequency, duration, and repetition rate (Liu et al., 2003). These data from rodents thus suggest that, irrespective of the capacity for vocal learning, age-dependent reductions in the acoustic variability of vocalizations are likely a natural part of vocal development.

Why pups emit USVs has been a question of long-standing interest in rodent bioacoustics, with few studies investigating the communicative function of prairie vole pup USVs in particular. One hypothesized driver of vocal emission in other rodent species is pup temperature. Moving young pups to a colder location reliably evokes USVs (Okon, 1970, 1971, 1972; Blake, 1992), leading mothers to retrieve pups back to the warmth of the nest (Sewell, 1970; Shapiro and Insel, 1990; Brunelli et al., 1994; Ehret, 2005; Bowers et al., 2013). Kept at a thermoneutral temperature, however, relocated pups do not increase their vocal rate (Allin and Banks, 1971). The influence of external temperature on vocal activity diminishes as pups achieve thermoregulatory competence, with older pups exhibiting no vocal response to temperature changes (Okon, 1970). In our study of prairie voles, however, pups vocalized even after they reached an age when thermoregulatory competence was achieved. Thus, while undeveloped thermoregulation may explain the vocal emission of young pups, vocalizations from older pups must be differentially driven.

A second hypothesis in the field is that USV emission indicates a pup's emotional state (Noirot and Pye, 1969; Hahn et al., 1998; Ehret, 2005; Hahn and Lavooy, 2005; Portfors, 2007). Social isolation is a known stressor for prairie vole pups, leading to a hormonal stress response and a correlated increase in vocal emission (Shapiro and Insel, 1990). However, rodent pups consistently vocalize more upon experiencing a second isolation from their mother than when they are first isolated (Hofer et al., 1998; Shair, 2014; Robison et al., 2016). These maternally potentiated vocalizations cannot be a direct response to the immediate environmental conditions, since they are identical. Instead, these experiences may produce different stress responses, leading to distinct vocal patterns. Additionally, drugs that increase stress also increase USV production, while stress-reducing drugs diminish vocal emission (Hahn and Lavooy, 2005; Costantini and D'amato, 2006). Thus, instead of reflecting acute physical conditions, vocal activity may provide an accurate readout of the internal state experienced by pups.

In our study, we manipulated social context to impact prairie vole pups' internal states. We placed a pup's mother on the opposite side of a transparent barrier and characterized the pup's vocal responses. While rodent pups exhibit olfactory competence as early as P2 (Cornwell-Jones and Sobrian, 1977; Geyer, 1979), ear and eye opening occur during our window of observation. Young pups (P8) significantly decreased their vocal emissions when their mother was introduced. This may be due to habituation to the environment, as vocalization levels consistently decreased over 11 min, then stabilized. Interestingly, only after pups were competent in both hearing and seeing their surroundings did they vocalize more after adding the mother. This increased calling cannot be attributed to a desire for warmth, as pups could thermoregulate by this age. While pups at all ages were fully covered in fur, older pups also had thicker fur, likely aiding their thermoregulation. Therefore, our results may indicate that older pups' stress levels increase when the mother is nearby but not physically accessible. Alternatively, assuming that pups vocalize to elicit contact from their mother, our results may reveal the developmental emergence of using vocalizations to satisfy the drive for social contact.

In fact, social drive becomes the most reliable modulator of rodent vocal emission by adulthood. For example, adult mice, while typically silent in isolation (Whitney et al., 1973), are highly vocal in social settings. Female mice vocalize when exposed to conspecifics of either sex (D'Amato and Moles, 2001; Neunuebel et al., 2015; Hoier et al., 2016), while males are most vocal toward sexually-receptive females (Sewell, 1970; Whitney et al., 1974; Nyby et al., 1979). Enhancing social drive via social isolation leads to increased vocal emission, with pairs of isolated females vocalizing four times more than group-housed pairs (Zhao et al., 2021). Adult prairie vole vocalizations also indicate social motivation, with males and females both vocalizing toward conspecifics, and males vocalizing more toward sexually receptive females than non-receptive females (Ma et al., 2014). Thus, vocal activity by adult prairie voles is a reliable readout of social environment and social drive.

Together, our study provides a thorough assessment of the acoustic development of prairie vole pup vocalizations and the development of social-vocal communication. We find that vocalizations become shorter and more stereotyped with age. Additionally, pups exhibit an age-dependent modification in vocal emission rates, but not acoustic features, based on social context. These results uncover a potential developmental regulation in pups' ability to modify vocal emission based on their social drive and social environment, with pups not increasing social vocalizations until after they can hear and see. Future work is necessary to explore the neural circuitry underlying social communication, including the brain regions and developmental influences involved in the control of vocal behavior.

Furthermore, given the unique behavioral repertoire of adult prairie voles and their importance in furthering our understanding of the neuromodulatory regulation of social behaviors, prairie vole pups provide a novel avenue for assessing the role of neuromodulators in early-life social bonds and the development of social communication. For instance, oxytocin can be involved in social motivation in rodent pups. Oxytocin knockout (OTKO) mouse pups (P10) take longer than wildtype pups to approach their mother following separation, and show no preference for their mother over other females [P15 (Ross and Young, 2009)]. Concomitantly, both OTKO (Winslow et al., 2000) and oxytocin-receptor knockout [OTRKO (Takayanagi et al., 2005)] mouse pups vocalize less during social isolation than wildtype pups, consistent with USVs as a readout for pups' social drive for maternal contact. Very young OTRKO prairie voles (P2–P5) have also been assessed for isolation-induced USV and found not to vocalize less (Horie et al., 2019), but this may reflect the influence of thermoregulation rather than social motivation at these ages, as our data suggest. Interestingly, rats selectively bred for lower USV emission exhibit increased oxytocin receptor expression in the nucleus accumbens (Brunelli et al., 2015). Thus, further work is necessary to determine the role of oxytocin in the development of social-vocal communication in prairie voles and the relationship between early-life oxytocinergic signaling and social-vocal communication during pair bonding in adulthood.

Finally, social-vocal communication is vital for social interaction and synchrony from birth. For instance, exposing human infants in the NICU to their mother's voice leads to enhanced wakefulness (Filippa et al., 2013) and can help ameliorate later deficits in mother-child interaction (Welch and Ludwig, 2017; Beebe et al., 2018). In other species, vocal communication enhances both behavioral and neural synchrony between individuals (Rose et al., 2021). Thus, while the possibilities of the prairie vole as a model of neurodevelopmental disorders and early life stressors are only beginning to be explored, work should begin to assess dyadic vocal interplay between pairs of animals. Our work sets the stage for future studies determining the neural and neurochemical bases of early-life social communication, and the impact of early-life manipulations on the development and use of social-vocal communication in rodent species.

## Data Availability Statement

The datasets present in this study can be found in a GitHub repository: https://github.com/rcbliu/Pup-USV-Data.

## Ethics Statement

Experiments were conducted in strict accordance with the guidelines established by the National Institutes of Health and then approved by Emory University's Institutional Animal Care and Use Committee.

## Author Contributions

AB, CF, and RL designed the study. CF, AB, MW, and DC developed the methodology. MW, DC, and AD produced all necessary software. MW and DC conducted formal analyses. AB, CF, DC, AD, and MW conducted the research. RL and LY provided all necessary resources. MW, DC, and RL wrote the original manuscript draft, with all authors providing reviews and edits. MW, DC, and RL generated data visualizations. LY, RL, CF, and AB acquired project funding. All authors contributed to the article and approved the submitted version.

## Funding

This work was supported by NIH grant P50MH100023 (LY and RL), R01MH115831 (RL and LY), 5R01DC008343 (RL), and P51OD11132 (YNPRC).

## Conflict of Interest

The authors declare that the research was conducted in the absence of any commercial or financial relationships that could be construed as a potential conflict of interest.

## Publisher's Note

All claims expressed in this article are solely those of the authors and do not necessarily represent those of their affiliated organizations, or those of the publisher, the editors and the reviewers. Any product that may be evaluated in this article, or claim that may be made by its manufacturer, is not guaranteed or endorsed by the publisher.
